# The Role of Exercise Doppler Echocardiography to Unmask Pulmonary Arterial Hypertension in Selected Patients with Systemic Sclerosis and Equivocal Baseline Echocardiographic Values for Pulmonary Hypertension

**DOI:** 10.3390/diagnostics11071200

**Published:** 2021-07-02

**Authors:** Loukianos S. Rallidis, Konstantina Papangelopoulou, Anastasia Anthi, Iraklis Tsangaris, Christos Varounis, Georgios Makavos, Dimitrios Konstantonis, Panagiotis Vlachoyiannopoulos, Stylianos E. Orfanos, Efstathios K. Iliodromitis

**Affiliations:** 1 Second Department of Cardiology and Pulmonary Hypertension Clinic, School of Medicine, National & Kapodistrian University of Athens, Attikon Hospital, 16462 Athens, Greece; kpapanel@gmail.com (K.P.); varounis@hotmail.com (C.V.); gmakavos@hotmail.com (G.M.); iliodromitis@yahoo.gr (E.K.I.); 2 Second Department of Critical Care and Pulmonary Hypertension Clinic, School of Medicine, National & Kapodistrian University of Athens, Attikon Hospital, 16462 Athens, Greece; anastasia.anthi1@gmail.com (A.A.); itsagkaris@med.uoa.gr (I.T.); dkonstantonis@gmail.com (D.K.); stylianosorfanosuoa@gmail.com (S.E.O.); 3 Department of Pathophysiology, School of Medicine, National & Kapodistrian University of Athens, 11527 Athens, Greece; pvlah@med.uoa.gr

**Keywords:** exercise Doppler echocardiography, pulmonary arterial hypertension, systemic sclerosis

## Abstract

Recently, a lower mean pulmonary arterial pressure (PAP) cutoff of >20 mmHg for pulmonary hypertension (PH) definition has been proposed. We examined whether exercise Doppler echocardiography (EDE) can unmask PA hypertension (PAH) in systemic sclerosis (SSc) patients whose baseline echocardiography for PH is equivocal. We enrolled 49 patients with SSc who underwent treadmill EDE. Tricuspid regurgitation (TR) velocity was recorded immediately after EDE. Inotropic reserve of right ventricle (RV) was assessed by the change (post-prior to exercise) of tissue Doppler imaging-derived peak systolic velocity (S) of tricuspid annulus. Inclusion criteria comprised preserved left and RV function, and baseline TR velocity between 2.7 and 3.2 m/s. All patients had right-heart catheterization (RHC) within 48 h after EDE. From 46 patients with good quality of post-exercise TR velocity, RHC confirmed PAH in 21 (45.6%). Post-exercise TR velocity >3.4 m/s had a sensitivity of 90.5%, a specificity of 80% and an accuracy of 84.8% in detecting PAH. Inotropic reserve of RV was positively correlated with maximum achieved workload in METs (*r* = 0.571, *p* < 0.001). EDE has a good diagnostic accuracy for the identification of PAH in selected SSc patients whose baseline echocardiographic measurements for PH lie in the gray zone, and it is also potentially useful in assessing RV contractile reserve.

## 1. Introduction

Systemic sclerosis (SSc) is an autoimmune rheumatic disease commonly complicated by pulmonary hypertension (PH) [1]. In most cases, PH in SSc is due to pulmonary arterial hypertension (PAH) [2]. The prevalence of PAH in SSc ranges from 5% to 12% [3] and it is the leading cause of death in these patients [4,5].

Early diagnosis of PH in SSc is of critical importance, not only because of the rapid progression of the disease but also because it can lead to timely initiation of treatment [6] and improvement of survival, particularly in patients under the age of 70 years [7]. It has been indicated by randomized controlled studies that treatment of mildly symptomatic patients with PAH is effective in terms of improvement in exercise capacity, functional class, hemodynamics, echocardiographic parameters associated with PH and delay of clinical worsening [8]. While the “gold standard” for PH diagnosis remains the measurement of pulmonary arterial pressure (PAP) by right-heart catheterization (RHC) [3], resting echocardiography is the most useful tool for PH screening, as it provides a noninvasive assessment of systolic PAP (sPAP) using tricuspid regurgitation (TR) velocity, which correlates well with invasive measurements [9]. However, resting echocardiography is of limited accuracy for the detection of elevated PAP in SSc, particularly if TR velocity is <3.4 m/s [10]. SSc patients often complain of dyspnea on exertion without echocardiographic evidence of PH at rest [11].

Exercise Doppler echocardiography (EDE) in SSc patients has been proposed as a useful technique for detection of elevations in sPAP during exercise, suggestive of subclinical PAH due to progressive remodeling and functional abnormalities of the pulmonary arterial vasculature [11,12]. However, the role of EDE in the detection of exercise-induced PH has been downgraded in the 2015 ESC/ERS guidelines for the diagnosis and treatment of PH because of limited standardization and a lack of prospective confirmatory data [3,13]. However, exercise echocardiography can provide additional information beyond changes in sPAP by assessing the exercise tolerance, the right ventricular (RV) contractile reserve and the left ventricular (LV) diastolic function [14].

Recently, the cutoff value for the definition of PH was set at the level of mean PAP > 20 mmHg, measured by RHC [15]. This challenged us to evaluate the potential usefulness of EDE to unmask the presence of PAH in selected asymptomatic or mildly symptomatic patients with SSc, whose resting echocardiographic sPAP fall within the “gray zone” (sPAP = 35–45 mmHg, which corresponds to TR velocity = 2.7–3.2 m/s).

## 2. Methods

### 2.1. Study Population

We prospectively recruited consecutive patients with established SSc who were referred to the PH clinic of our center for screening for PAH. Patients seen in the PH clinic routinely undergo a detailed clinical work-up which includes resting echocardiogram, 6-minute walk test (6-MWT), pulmonary function tests and laboratory tests, including N-terminal pro-brain natriuretic peptide (NT-proBNP).

Inclusion criteria for our study comprised: (1) preserved left ventricular (LV) function (ejection fraction (EF) > 55%) and right ventricular (RV) function (tricuspid annulus plane systolic excursion (TAPSE) ≥ 16 mm), (2) no history of previous ischemic or valvular heart disease or echocardiographic evidence of diastolic dysfunction consistent with elevated LV filling pressure, (3) presence of TR with a good-quality signal on Doppler, (4) sinus rhythm, (5) baseline maximal TR velocity in the range of 2.7–3.2 m/s and (6) forced expiratory volume in 1 s (FEV_1_) ≥ 55% of predicted normal and total lung capacity (TLC) ≥ 60% of predicted normal [16].

The study was approved by the ethics committee of our institution and all subjects provided signed informed consent. The study was conducted according to the guidelines of the Declaration of Helsinki, and approved by the Ethics Committee of University General Hospital Attikon. Approval number: EΒΔ36. Date of approval 14 February 2014. 

### 2.2. Resting Echocardiography

A comprehensive resting echocardiography study was performed during the visit to the PH clinic with a Vivid 9 ultrasound system (GE Medical Systems, Horten, Norway). All echocardiographic studies were performed by a single operator (L.S.R.) and the measurements were performed according to the current guidelines of the American Society of Echocardiography and the European Association of Cardiovascular Imaging [17]. Assessment of diastolic function was based on pulsed-wave Doppler of transmitral flow E and A waves and deceleration time. Tissue Doppler imaging (TDI) pulsed-wave velocities in early diastole (e’ wave) were recorded at the lateral mitral annulus aspect and septal basal regions from the apical four-chamber view [18]. The average E/e’ ratio was calculated by taking the mean of lateral and septal e’ waves. Markers of RV function, such as TAPSE, and TDI-derived peak systolic velocity (S) of the tricuspid annulus (S(RV)) were also recorded. TR velocity was measured with continuous-wave Doppler. Right atrial pressure (RAP) was calculated from the inferior vena cava (IVC) diameter and its respiratory variation [19]. sPAP was calculated according to the Bernoulli equation: sPAP = 4 × (TR velocity)^2^ + RAP.

A repeat resting echocardiographic study was performed the day of RHC. For comparisons of echocardiographic with RHC measurements, the echocardiographic values obtained the day of RHC were used.

### 2.3. Exercise Doppler Echocardiography

EDE was performed using a modified Bruce protocol. The modified protocol was preferred over the standard because patients unfamiliar with treadmill (usually elderly women) were more compliant to perform this “softer” protocol. Exercise was terminated when patients achieved 85% of the predicted maximal heart rate or until their exercise capacity was limited by symptoms, i.e., symptoms-limited exercise test. Echocardiographic images were acquired prior to (special attention was paid to accurate measurement of maximal TR velocity) and immediately after conclusion of the treadmill exercise. In particular, upon completion of the study, the patients returned to the bed which was situated beside the treadmill machine at lateral decubitus position, and TR velocity was first measured followed by evaluation of RV systolic function and LV diastolic function from the four-chamber view. All measurements were obtained within 1 min by a single operator (L.S.R.) with expertise in EDE [20]. RV systolic exercise reserve was defined by the difference (denoted by “Δ”) between post-exercise and respective baseline values of TAPSE and S(RV). Additional measurements included resting heart rate and blood pressure (BP), as well peak heart rate and BP, total time of exercise and maximum workload estimated by standard metabolic equivalents (METs).

### 2.4. Right-Heart Catheterization

All participants underwent RHC in a supine position using an echo-guided right internal jugular vein access. The following measurements were performed: RAP, PAP (systolic, diastolic and mean), pulmonary arterial wedge pressure (PAWP) and cardiac output (CO) by thermodilution. Pulmonary vascular resistance (PVR) was subsequently calculated.

### 2.5. 6-Minute Walk Test

Patients performed a 6-MWT under the guidance and supervision of an expert nurse. A pre-marked corridor was used, and total walking distance was measured as well as percutaneous oxygen saturation at the end of the test.

### 2.6. Pulmonary Function Tests and Lung Imaging

All patients underwent chest-XR, high-resolution computed tomography (CT) of lungs and pulmonary function tests including FEV_1_ and TLC.

### 2.7. Biochemical Measurements

Blood was taken from all patients for NT-proBNP and high-sensitivity C-reactive protein (hsCRP) measurements, as well as for routine hematological and biochemical tests.

### 2.8. Statistical Analysis

Continuous variables are presented as means ± standard deviation (SD), while non-normally distributed variables are presented as medians and interquartile ranges. The Student’s t test was applied for independent samples to compare means for normally distributed variables or the Mann–Whitney test for skewed variables.

Cutoff analysis using receiver operating characteristic (ROC) analysis revealed the optimal cutoff value with the best combination of sensitivity and specificity that discriminates patients according to whether they had PAH or not. Sensitivity, specificity, disease prevalence, positive and negative predictive value as well as accuracy are expressed as percentages. Confidence intervals (CIs) for sensitivity, specificity and accuracy are “exact” Clopper–Pearson CIs. CIs for the likelihood ratios are calculated using the “Log method” [21]. CIs for the predictive values are the standard logit CIs [22].

A *p*-value < 0.05 was considered significant. The SPSS version 26 (SPSS Inc., Chicago, IL, USA) statistical package was used.

## 3. Results

During the period 2016–2020, 52 out of 190 consecutive patients with SSc referred to the PH clinic fulfilled the inclusion criteria. Of those, 3 had a poor-quality TR Doppler signal in post-exercise echocardiography and 3 were unable to perform the treadmill exercise due to severe musculoskeletal problems. Thus, the final study group comprised 46 patients with SSc. Of those, 37 (80%) had diffused cutaneous SSc and 9 (20%) had limited cutaneous SSc (CREST syndrome). The majority of patients (44 of 46) were women. The mean disease duration from first symptoms related to SSc at the time of the first visit was 7.4 ± 5.1 years (Table 1).

RHC confirmed the presence of PAH (mean PAP > 20 mmHg, PAWP < 15 mmHg and PVR > 3 Wood Units) in 21 patients (45.6%). With the previous cutoff level of mean PAP > 25 mmHg, only 10 patients (21.7%) would have been classified in the PAH group. Resting sPAP estimated by echocardiography the day of RHC was positively corelated with sPAP measured by RHC (*r* = 0.658, *p* < 0.001).

Table 1 shows patients’ features according to the presence of PAH. Patients with PAH achieved higher post-exercise TR velocity, had lower RV inotropic response (indicated by lower Δ (S(RV)) and Δ (TAPSE)) and poorer exercise capacity (achieved METs) compared with patients without PAH. There was no difference between the two groups in NT-proBNP and hsCRP levels as well in the post-exercise ratio E/e’.

ROC curve analysis of post-exercise TR velocity discriminated the patients according to those with RHC-confirmed PAH and those without (AUC = 0.927, with 95%CI: 0.856–0.997, *p* < 0.001). A cutoff value of post-exercise TR velocity > 3.4 m/s had a sensitivity of 90.5%, a specificity of 80% and an accuracy of 84.8% in detecting PAH validated by RHC (Table 2). The selected cutoff value had the highest combination of sensitivity and specificity. In addition, ROC curve analysis of the difference “(post-exercise)–(prior to exercise) TR velocity” discriminated the patients according to those with RHC-confirmed PAH and those without (AUC = 0.834, with 95%CI: 0.714–0.955, *p* < 0.001). A cutoff value of the difference of “(post-exercise)–(prior to exercise) TR velocity” > 0.5 m/s had a sensitivity of 90.5%, a specificity of 64% and an accuracy 76% in detecting PAH validated by RHC (Table 3).

**Figure 1:**
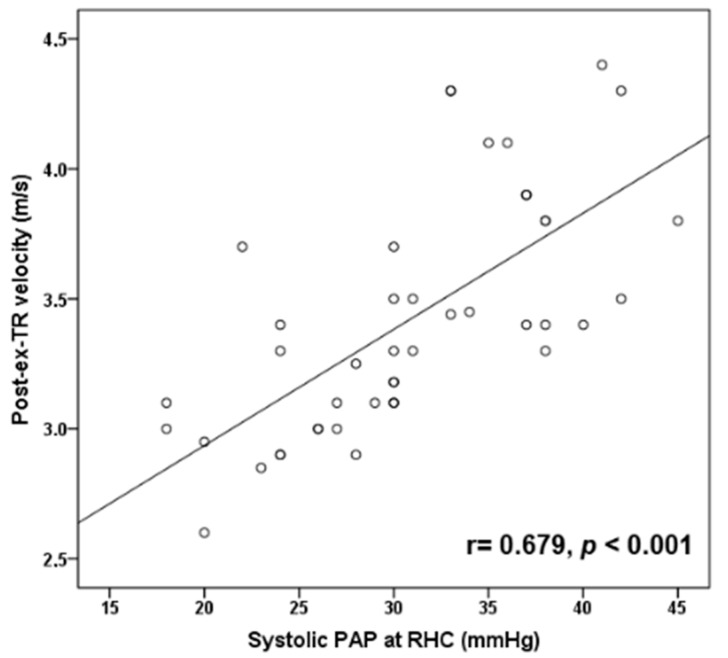
Scatter plot correlation of post-exercise tricuspid regurgitation velocity (post-ex-TR velocity) with resting systolic pulmonary arterial pressure (PAP) at right-heart catheterization (RHC).

**Figure 2:**
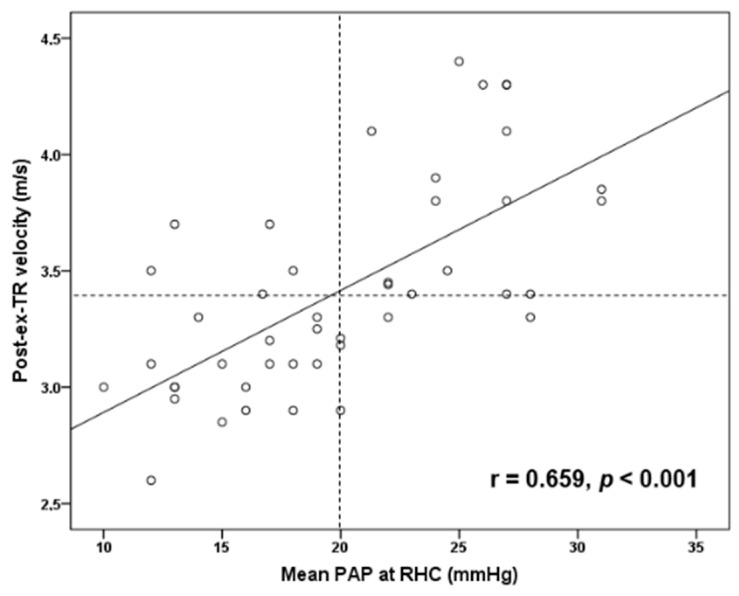
Scatter plot correlation of post-exercise tricuspid regurgitation velocity (post-ex-TR velocity) with resting mean pulmonary arterial pressure (PAP) at right-heart catheterization (RHC). Vertical dashed line indicates mean PAP of 20 mmHg, while horizontal dashed line indicates post-ex-TR velocity of 3.4 m/s.

**Figure 3:**
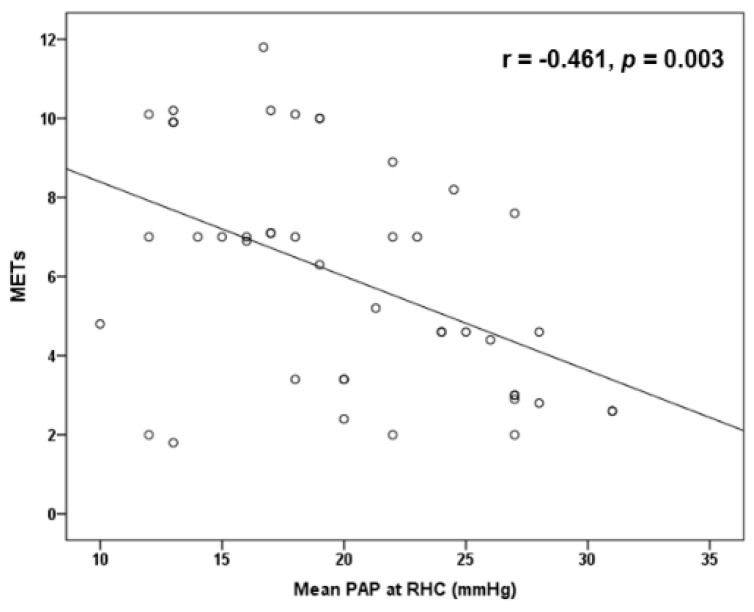
Scatter plot inverse correlation of maximal achieved workload in METs with resting mean pulmonary arterial pressure (PAP) at right-heart catheterization (RHC).

**Figure 4:**
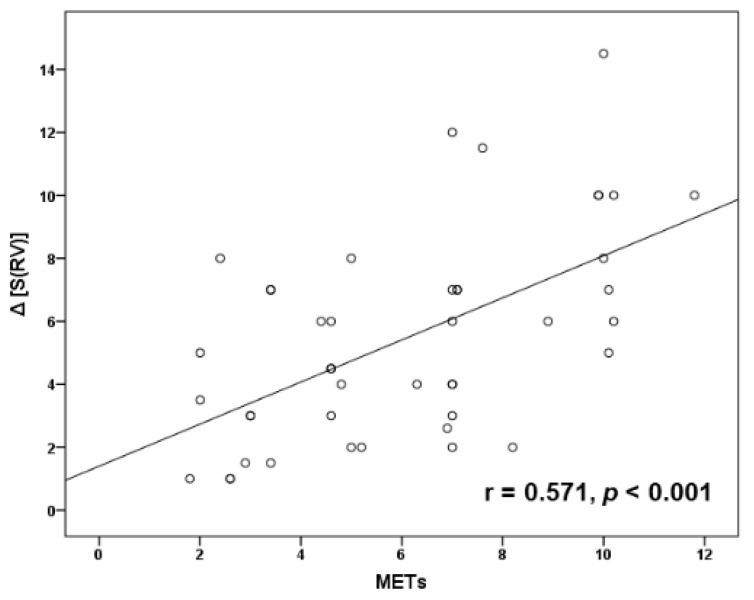
Scatter plot correlation of maximal achieved workload in METs with right ventricular (RV) inotropic reserve defined by the difference between post-exercise and prior to exercise S wave of RV, i.e., Δ (S(RV)).

Post-exercise TR velocity was positively correlated with resting sPAP (*r* = 0.679, *p* < 0.001) (Figure 1) and mean PAP (*r* = 0.659, *p* < 0.001) (Figure 2) obtained by RHC, while maximal achieved workload in METs was inversely correlated with mean PAP (*r* = −0.461, *p* = 0.003) (Figure 3) and PVR (*r* = −0.428, *p* = 0.005). In addition, exercise systolic inotropic reserve of RV assessed by Δ (S(RV)) was positively correlated with METs (*r* = 0.571, *p* < 0.001) (Figure 4) and with 6-MWT (*r* = 0.436, *p* = 0.01), while Δ (TAPSE) did not show similar correlations (*p* > 0.05).

## 4. Discussion

In this study, we showed that EDE is a useful tool for the early detection of PAH in selected asymptomatic or mildly symptomatic patients with SSc whose baseline echocardiographic measurements for PH fall in the gray zone. In addition, exercise-induced RV contractile response was correlated with both patients’ achieved exercise workload and their functional capacity, as it is reflected by the 6-MWT.

In particular, we found that post-exercise TR velocity > 3.4 m/s, which corresponds to a sPAP ≈ 50 mmHg, had a good diagnostic accuracy for the identification of PAH validated by RHC. Our study is the first to address this issue by adopting the recently proposed cutoff value of mean PAP > 20 mmHg for the diagnosis of PAH measured by RHC. This new value has already been endorsed by the 2020 guidelines for the management of adult congenital heart disease [23].

There are few data assessing the sensitivity and specificity of EDE in identifying PAH confirmed by RHC in patients with SSc [12,24]. Steen et al. [12] reported that among 54 patients with SSc and clinical characteristics of high risk for PAH, 44% had a positive treadmill exercise test, defined as an increase in sPAP ≥ 20 mmHg over the resting rate, and RHC confirmed PH in 81% of them. Baptista et al. [13] in 2016 reviewed 15 studies which enrolled 1242 patients with SSc who performed EDE. The weighted mean sPAP on exercise was 43 mmHg, the mean increase in sPAP was 15 mmHg and more than half of the studies reported mean exercise sPAP ≥ 40 mmHg. However, it was not possible for the authors to reach definite conclusions on the value of EDE due to the great heterogeneity in the methods, the characteristics of the recruited populations, the protocols and the definition of the positive results.

This great heterogeneity does not allow us to compare our results with previous studies. We recruited asymptomatic or oligosymptomatic patients with SSc whose echocardiographic findings were equivocal for PH. In addition, we applied the recently proposed cutoff value of mean PAP > 20 mmHg for the definition of PH, which has not been applied in other studies.

Previous studies have reported a high prevalence of exercise-induced PH in SSc patients. Using cutoff values of sPAP > 40 or >50 mmHg, 40–60% of patients with SSc develop exercise-induced PH [25,26,27,28]. However, the interpretation of sPAP elevations in the setting of SSc is complex since it is multifactorial, and its exact natural history is unknown [28]. There are several determinants of exercise-induced PH in SSc patients, such as reduced pulmonary vascular reserve due to early pulmonary arterial vasculature remodeling, LV diastolic dysfunction and lung interstitial fibrosis.

We selected patients without systolic or prominent diastolic LV dysfunction or abnormal pulmonary function tests. By minimizing the contribution of left heart disease (group 2 PH) or lung disease (group 3 PH) in the development of PH, exercise-induced elevations in sPAP are likely to be caused mainly by abnormal pulmonary vascular response and represent mostly an early phase of PAH. In our study, the minor impact of diastolic function in the elevation of post-exercise sPAP is supported by the lack of increase of post-exercise E/e’ > 15, a value that has been set as the threshold for pathologically elevated PAWP [29]. A number of studies have shown a good correlation between exercise E/e’ with PAWP simultaneously measured with RCH in patients with preserved LV function [30,31].

Another critical component of exercise-induced PH is the magnitude of CO increase. In normal subjects, sPAP increases to 34.3 ± 7.5 mmHg, in parallel to large elevations in CO (≈20 L/min) [32]. However, in SSc, sPAP is disproportionally elevated in relation to concomitant elevations of CO during exercise. It has been reported that for a significantly lower CO (<8–10 L/min) achieved in SSc studies, sPAP increased to approximately 47 mmHg [13]. The relatively small CO increase during exercise in SSc may be explained by limited exercise capacity due to interstitial lung fibrosis, PVD, osteoarticular issues or deconditioning [13]. Therefore, interpretation of exercise-induced increases in sPAP should ideally be performed in relation to blood flow changes. ΔPAP/ΔCO slopes reflecting pulmonary vascular reserve calculated by measurement of continuous pressure-flow values during exercise seem to be a more accurate determinant of exercise-induced PH in SSc [33]. Therefore, performance of exercise with parallel RHC may be meaningful in SSc patients with normal resting sPAP for prognostic stratification since it has been shown that steeper increases in mean PAP/CO slope are predictive of future development of PH [34]. However, this approach is invasive and cannot easily be applied in daily clinical practice as a screening test.

Our hypothesis is that in early stages of PVD in SSc patients, resting sPAP may be normal or slightly elevated due to pulmonary vasculature reserve. However, pulmonary flow augmentation during exercise in the context of inadequate adaptation of pulmonary vasculature due to impaired vascular distensibility may lead to a hemodynamic derangement, with a subsequent disproportionate increase in mean PAP [35].

We found that SSc patients with PAH had less RV inotropic reserve with exercise compared to those without PAH, and that exercise-induced contractile response was correlated with both patients’ achieved exercise workload and functional capacity. Interestingly, this association was found when RV inotropic response was assessed with Δ (S(RV)) but not with Δ (TAPSE). Although both measures represent longitudinal contraction of RV at the level of tricuspid annulus, it has been suggested that S wave may be more sensitive than TAPSE to assess RV inotropic response [36]. We also found a positive correlation of post-exercise TR velocity with resting mean PAP and sPAP obtained by RHC, while maximum workload was inversely correlated with mean PAP. The latter has been reported previously [11] and it is reasonable to speculate that the higher mean resting PAP is associated with higher PVR during exercise, with subsequent reduced carbon dioxide exchange, increased anaerobic metabolism, muscle fatigue and reduction in exercise capacity.

Of note, there was no difference in the 6-MWT between SSc patients with PAH and those without PAH. This may be explained by a “ceiling effect” of the 6-MWΤ which may mask the disability of less-symptomatic PAH patients. Therefore, the 6-MWΤ was relatively preserved in our PAH patients who were asymptomatic or mildly symptomatic, probably due its low sensitivity to reveal functional impairment in this subgroup of PAH patients [37].

It should be mentioned that EDE may not be applicable in all asymptomatic or oligosymptomatic SSc patients, since (a) a small proportion of patients (6% in our study) may not be able to perform a treadmill exercise test due to severe myalgias, arthralgias and musculoskeletal problems being part of their disease process, and (b) not all patients undergoing EDE have a reliably measurable TR velocity immediately post-exercise. In our study, 6% of our patients had a poor-quality Doppler signal, and thus were excluded from further analysis.

### Study Limitations

The main limitation of this study is the relatively small sample size.

## 5. Conclusions

EDE has a good diagnostic accuracy for the identification of PAH in selected asymptomatic or oligosymptomatic patients with SSc, whose baseline echocardiographic measurements for PH lie in the gray zone. Thus, it might be useful to perform EDE in SSc with equivocal clinical and echocardiographic findings of PH, and if abnormal, to proceed to RHC. Furthermore, EDE provides information regarding the inotropic reserve of RV, but its clinical usefulness and prognostic value need to be investigated in prospective studies.

## Figures and Tables

**Table 1:** Characteristics of patients with systemic sclerosis according to the presence of pulmonary arterial hypertension (PAH).

Characteristics	All Patients	Patients with PAH (*n* = 21)	Patients without PAH (*n* = 25)	*p*-Value *
Age (years)	61.4 ± 10.7	57.8 ± 9.5	62.9 ± 10.6	0.087
Body mass index (kg/m^2^)	25.7 ± 4.3	25.5 ± 5.8	25.8 ± 2.7	0.805
Duration of disease (years)	7.4 ± 5.1	5.9 ± 4.1	8.0 ± 5.4	0.257
FEV_1_, % predicted	75.6 ± 17.8	73.5 ± 16.4	78.4 ± 19.9	0.529
TLC, % predicted	75.6 ± 7.1	76.3 ± 7.1	74.5 ± 7.1	0.480
6-minute walk test (m)	452.2 ± 72.9	456.8 ± 72.9	459.2 ± 66.7	0.917
Baseline echocardiography (echo)				
Ejection fraction of left ventricle (%)	63.8 ± 3.4	63.1 ± 3.7	64.3 ± 3.1	0.228
E/e’ (average)	10.1 ± 2.3	9.3 ± 1.7	10.6 ± 2.6	0.068
Left atrial index (mL/m^2^BSA)	27.3 ± 3.9	26.8 ± 4.5	27.6 ± 3.7	0.523
TAPSE (mm)	22.4 ± 4.3	21.3 ± 3.3	24.0 ± 3.4	0.012
S (RV) (cm/s)	13.5 ± 2.9	12.3 ± 2.9	14.8 ± 2.2	<0.001
Right atrial pressure (mmHg)	3.8 ± 1.8	4.5 ± 2.1	3.2 ± 1.0	0.011
TR velocity (m/s)	2.85 ± 0.19	2.92 ± 0.16	2.74 ± 0.08	<0.001
sPAP (mmHg)	35.4 ± 4.1	38.2 ± 4.2	32.9 ± 2.1	<0.001
Right-heart catheterization				
Right atrial pressure (mmHg)	4.2 ± 2.0	4.8 ± 2.3	3.8 ± 1.9	0.157
PAWP (mmHg)	7.3 ± 2.3	7.1 ± 2.1	7.5 ± 2.6	0.552
Mean PAP (mmHg)	19.5 ± 5.6	25.6 ± 2.9	15.9 ± 3.0	<0.001
sPAP (mmHg)	30.6 ± 6.8	37.3 ± 3.6	25.8 ± 3.9	<0.001
Cardiac output (L/min)	5.0 ± 1.3	4.45 ± 0.92	5.45 ± 1.21	0.005
PVR (Woods Units)	2.63 ± 1.49	4.21 ± 0.97	1.58 ± 0.42	<0.001
Treadmill exercise				
Duration (min)	6.8 ± 3.7	5.15 ± 3.2	8.26 ± 3.7	0.005
Peak				
Heart rate (b/min)	141.4 ± 18.1	140.1 ± 13.8	141.7 ± 21.1	0.770
METs	6.1 ± 2.9	4.6 ± 2.1	7.0 ± 3.0	0.004
sBP (mmHg)	150.0 ± 25.4	139.7 ± 21.6	160.3 ± 22.9	0.006
Immediate post-exercise echo				
E/e’ (average)	9.1 ± 2.2	8.6 ± 2.1	9.5 ± 2.2	0.175
TR velocity (m/s)	3.4 ± 0.44	3.77 ± 0.36	3.14 ± 0.26	<0.001
sPAP (mmHg)	51.1 ± 12.9	61.57 ± 10.97	42.65 ± 7.09	<0.001
TAPSE (mm)	26.9 ± 5.1	24.6 ± 4.4	29.4 ± 4.0	<0.001
S(RV) (cm/sec)	18.7 ± 4.4	16.7 ± 4.2	21.2 ± 4.4	0.001
Δ (TAPSE) (mm)	4.5 ± 2.8	3.4 ± 2.0	5.4 ± 3.0	0.017
Δ (S(RV)) (cm/s)	5.5 ± 3.3	4.3 ± 3.2	6.3 ± 3.1	0.043
Biochemistry				
hsCRP (mg/L)	3.2 (3.1–5.1)	3.35 (3.1–7.1)	3.11 (3.1–4.8)	0.462
NT-proBNP (pg/mL)	190.1 ± 169.4	177.9 ± 166.8	208.4 ± 169.2	0.556
Creatinine (mg/dL)	0.80 ± 0.21	0.75 ± 0.14	0.83 ± 0.25	0.227
Hematocrit (%)	38.9 ± 2.4	39.0 ± 1.8	38.85 ± 3.1	0.864

* comparison between PAH patients with those without PAH, FEV_1_ = forced expiratory volume in 1 s, TLC = total lung capacity, BSA = body surface area, PVR = pulmonary vascular resistance, sPAP = systolic pulmonary arterial pressure, PAWP = pulmonary arterial wedge pressure, TAPSE = tricuspid annular plane systolic excursion, TR = tricuspid regurgitation, NT-proBNP = N-terminal-pro brain natriuretic peptide, METs = metabolic equivalents (1 MET = 1 kcal/kg/h), RV = right ventricle, hsCRP = high-sensitivity C-reactive protein, S = systolic velocity of the tricuspid annulus, sBP = systolic blood pressure.

**Table 2:** Diagnostic test evaluation of the post-exercise tricuspid regurgitation velocity > 3.4 m/s.

	Point Estimate	95%CI
Sensitivity (%)	90.5	69.6–98.8
Specificity (%)	80.0	59.3–93.1
LR+	4.52	2.0–10.0
LR–	0.12	0.03–0.45
PPV (%)	79.1	63.1–89.3
NPV (%)	90.9	72.5–97.4
Accuracy (%)	84.8	71.1–93.6

CI = confidence interval, LR+ = positive likelihood ratio, LR– = negative likelihood ratio, PPV = positive predictive value, NPV = negative predictive value.

**Table 3:** Diagnostic test evaluation of the difference “(post-exercise)–(prior to exercise) tricuspid regurgitation velocity” > 0.5 m/s.

	Point Estimate	95%CI
Sensitivity (%)	90.5	69.6–98.8
Specificity (%)	64.0	42.5–82.0
LR+	2.51	1.46–4.32
LR–	0.15	0.04–0.57
PPV (%)	67.8	55.09–78.3
NPV (%)	88.9	67.5–96.8
Accuracy (%)	76.0	61.2–87.4

CI = confidence interval, LR+ = positive likelihood ratio, LR– = negative likelihood ratio, PPV = positive predictive value, NPV = negative predictive value.

## Data Availability

The data presented in this study are available on reasonable request from the corresponding author (L.S.R.). The data are not publicly available due to privacy and ethical issues.
